# Influence of a Sustained Release Deslorelin Acetate Implant on Reproductive Physiology and Associated Traits in Laying Hens

**DOI:** 10.3389/fphys.2018.01846

**Published:** 2018-12-20

**Authors:** Beryl Katharina Eusemann, Ahmad Reza Sharifi, Antonia Patt, Ann-Kathrin Reinhard, Lars Schrader, Christa Thöne-Reineke, Stefanie Petow

**Affiliations:** ^1^Institute of Animal Welfare and Animal Husbandry, Friedrich-Loeffler-Institut, Celle, Germany; ^2^Department of Animal Sciences, Georg-August-Universität Göttingen, Göttingen, Germany; ^3^Institute of Animal Welfare, Animal Behavior and Laboratory Animal Science, Fachbereich Veterinärmedizin, Freie Universität Berlin, Berlin, Germany

**Keywords:** laying hen, egg, follicle, gonadotropin-releasing hormone, deslorelin acetate, estradiol, keel bone, foot pad dermatitis

## Abstract

The aim of this study was to develop an animal model with non-laying hens which would allow for investigation of the relationship between egg production and common diseases in hens. A total of 40 Lohmann Selected Leghorn hens were kept for 20 weeks in a floor housing system in two groups: group “Adult” (21 weeks of age) and group “Juvenile” (14 weeks of age). In each group, 10 hens were administered a 4.7 mg sustained release deslorelin acetate implant subcutaneously; in group “Adult” after, in group “Juvenile” before the onset of lay. In both groups, the remaining hens served as control hens. An examination of each hen was performed weekly, including ultrasonography to check for ovarian follicles, analysis of estradiol-17ß plasma concentration, and assessment of comb size. Digital radiographs of the keel bone were taken in experimental weeks 7 and 15. No follicles were detected on the ovary of treated hens for a certain time period which varied between individuals (between 8 weeks and until the end of the experiment). Estradiol-17ß concentrations were significantly higher in control hens. The comb was significantly smaller in treated hens. A lower prevalence of keel bone damage (group “Adult”) and foot pad dermatitis (FPD) (both groups) was found in treated compared to control hens. These results show that a model with laying and non-laying hens can be achieved by administering a deslorelin acetate implant. Furthermore, they indicate a relationship between egg production and keel bone damage as well as FPD.

## Introduction

Laying hens often suffer from a variety of diseases such as osteoporosis (FAWC, 2010), keel bone fractures and deviations (Fleming et al., 2004; Rodenburg et al., 2008; Käppeli et al., 2011b; Wilkins et al., 2011; Petrik et al., 2015), and fatty liver hemorrhagic syndrome (Shini and Bryden, 2009). The high laying performance might be a related factor to these medical conditions. Concerning bone diseases, it is known that there is a high calcium demand for the egg shell and thus high laying performance may lead to weaker bones (Whitehead et al., 2003; FAWC, 2010). Concerning the fatty liver hemorrhagic syndrome, high performing hens may be more susceptible because of the stimulation of lipogenesis in the liver during egg production (Butler, 1976; Aydin, 2005; Deng et al., 2012).

A promising way to investigate possible relationships between egg production and these common diseases in laying hens as well as the underlying mechanisms may be the comparison of laying and non-laying hens of the same breed and age. Therefore, the aim of the current study was to develop an animal model in which egg production was selectively suppressed in hens to allow comparisons of traits with laying control hens.

An agent which is often used for reproductive management in several species is the synthetic gonadotropin-releasing hormone (GnRH) agonist deslorelin acetate. Naturally produced GnRH is secreted by the hypothalamus in a pulsatile manner (Carmel et al., 1976; Tsutsumi and Webster, 2009) and acts at pituitary receptors to induce the secretion of follicle-stimulating hormone (FSH) and luteinizing hormone (LH) (Ottinger et al., 2002). FSH and LH act at the gonads where they induce gametogenesis, gonadal steroidogenesis and, in females, ovulation. In contrast to pulsatile secretion, continuous presence of GnRH or its agonist results in a desensitization of the GnRH receptors and, consequently, a shutdown of the reproductive cascade (Belchetz et al., 1978; Rabin and McNeil, 1980; Ottinger et al., 2002; Gobello, 2007). This can be achieved by the use of slow-release deslorelin acetate implants, for example Suprelorin^®^ (Virbac, Carros, France). This implant has been developed for chemical castration in male dogs and is available in two different strengths: 4.7 mg deslorelin acetate and 9.4 mg deslorelin acetate. In dogs, the duration of effectiveness, i.e., the time period during which reproductive function is suppressed, is six (4.7 mg) and twelve (9.4 mg) months, respectively (Ponglowhapan, 2011). In pet birds, deslorelin acetate implants are frequently used to suppress undesired reproductive activity (Keller et al., 2013; Mans and Pilny, 2014). In adult female cockatiels, one 4.7 mg deslorelin acetate implant has been shown to significantly prevent egg laying for at least 180 days (Summa et al., 2017). Investigating the effect of deslorelin acetate on egg production in poultry, most studies have been conducted using Japanese quail. Petritz et al. (2013) found a complete suppression of egg production by a 4.7 mg deslorelin acetate implant in six out of ten hens for 10 weeks. Plasma concentration of estradiol-17ß was significantly lower in non-laying compared to laying quail. However, the implant did not show any effect on the laying activity in the remaining hens (Petritz et al., 2013). In a subsequent study, the authors found seven out of ten Japanese quail without oviposition in two treatment groups: one group was simultaneously treated with two deslorelin acetate 4.7 mg implants; the other group was treated with a single deslorelin acetate 9.4 mg implant. Egg production was decreased for approximately 14 weeks in the group with two deslorelin acetate 4.7 mg implants. In the group with one deslorelin acetate 9.4 mg implant, egg production was decreased for at least 14 weeks but there was a large variation between the birds (Petritz et al., 2015). In another study with Japanese quail, seven out of nine hens stopped laying eggs after the administration of a deslorelin acetate implant, the majority of them for more than 14 weeks (Schmidt et al., 2013). There is almost no knowledge about the effect of deslorelin acetate implants in chicken. Noonan et al. (2012) tested deslorelin acetate 4.7 mg and deslorelin acetate 9.4 mg in laying hens (*Gallus gallus*). Two weeks after implantation, all treated hens stopped laying eggs, regardless of the deslorelin acetate concentration. Suppression of egg production lasted almost 26 weeks in hens treated with deslorelin acetate 4.7 mg and 45.5 weeks in hens treated with deslorelin acetate 9.4 mg (Noonan et al., 2012). However, these results have to be interpreted with care because the hens were already 2 years old when being treated and the study was not published in a peer-reviewed journal but as a scientific abstract. Thus, detailed information about the study design and the analysis of the results is lacking.

Based on the results of these studies, we chose to use deslorelin acetate for the development of the desired animal model with non-laying and laying control hens in the current study. We tested whether a deslorelin acetate implant could suppress egg production in hens if implanted before or after the onset of lay. Furthermore, to characterize the animal model, we assessed whether the implant would lead to undesirable side effects and whether it would have any influence on other traits such as body weight, organ weight, general health, sexual hormone concentrations, and comb size.

## Materials and Methods

### Birds and Housing Conditions

The current experiment was performed in accordance with the German Animal Protection Law and approved by the Lower Saxony State Office for Consumer Protection and Food Safety (No. 33.19-42502-04-15/1966).

A total of 40 Lohmann Selected Leghorn (LSL) hens (*Gallus gallus domesticus)* were housed in two different age groups: one group (“Adult”, 20 hens) was 21 weeks at the beginning of the experiment whereas the other group (“Juvenile”, 20 hens) was 14 weeks old.

Table 1 details the age, management, and all experimental procedures for both groups in each experimental week.

**Table 1:** Management, experimental procedures, and age as well as equivalent implant week of the laying hens of group “Adult” and “Juvenile” in each experimental week.

Experimental week	Management	Experimental procedures	Group “Adult” (20 hens)		Group “Juvenile” (20 hens)	
			Week of age	Implant week	Week of age	Implant week
1	Arrival at experimental site		21	−3	14	−1
2		B+E	22	−2	15	0
3		B+E	23	−1	16	1
4	Relocation	B+E+U	24	0	17	2
5		B+E+U	25	1	18	3
6		B+E+U	26	2	19	4
7		B+E+U+R	27	3	20	5
8		B+E+U	28	4	21	6
9		B+E+U	29	5	22	7
10		B+E+U	30	6	23	8
11		B+E+U	31	7	24	9
12		B+E+U	32	8	25	10
13		B+E+U+CM	33	9	26	11
14		B+E+U+CM	34	10	27	12
15		B+E+U+CM+R	35	11	28	13
16		B+E+U+CM	36	12	29	14
17		B+E+U+CM	37	13	30	15
18		B+E+U+CM	38	14	31	16
19		B+E+U+CM	39	15	32	17
20		B+E+U+CM+D	40	16	33	18

*B, blood withdrawal for analysis of estradiol-17ß concentrations; E, health examination; U, ultrasonography of ovaries; CM, comb size measurement; R, radiography of keel bones; D, dissection; implant week 0, administration of deslorelin acetate implant; from this time point on, there were 10 treated and 10 control hens per group.*

All birds were obtained from a conventional breeding company (Zahrte, Wrestedt, Germany) at 14 weeks of age. In the breeding company, the animals had been reared under conventional conditions. After being brought to the experimental site, birds were kept in a floor housing system. During the first three experimental weeks, group “Adult” and group “Juvenile” were kept in two pens within the same poultry house that were separated by a solid wall. Each pen measured 11 m^2^, was littered with wood-shavings and straw and equipped with perches and a nest box measuring 0.54 m^2^. The light programs of both pens were independent from each other. The duration of the light period increased gradually from 10 h/day to 14 h/day. The 14 h light period was reached in the 24th week of age (group “Ault”) or in the 17th week of age (group “Juvenile”), respectively, and kept constant until the end of the experiment. Since hens of group “Juvenile” were exposed to 14 h light earlier than hens of group “Adult”, the two age groups were analyzed separately (see section Statistical Analysis). Light intensity was 20 lux at bird level.

In experimental week 4, all hens were relocated to another poultry house. Hens of both groups were housed in two pens which were separated by a fence, resulting in both groups being exposed to the same light program. Each pen measured 9 m^2^, was littered with wood-shavings and straw and equipped with perches and a nest box measuring 0.24 m^2^. The hens were offered water and a conventional complete feed for pullets (until the 18th week of age) and laying hens (from the 19th week of age on) *ad libitum*.

### Implantation of Deslorelin Acetate Implants

Ten hens of each group were given a 4.7 mg deslorelin acetate implant (Suprelorin^®^, Virbac, Carros, France). In group “Adult”, the implant was administered when all hens started laying (24th week of age; Table 1), whereas in group “Juvenile”, the implant was administered before the onset of lay (15th week of age; Table 1). The remaining ten hens of each group were kept as control hens within the same pen. The implant was administered subcutaneously between the scapulae. Hens were anaesthetized with 2–3% isoflurane (CP-Pharma Handelsgesellschaft mbH, Burgdorf, Germany) in compressed air with a flow rate of 500 ml/min delivered via face mask. Before application, the skin was aseptically prepared. After application, the implantation site was sealed with a tissue adhesive (Surgibond^®^, SMI, St. Vith, Belgium). Control hens did not receive any treatment.

### Weekly Health Examination of the Hens

The general health of all hens was checked daily and assessed weekly (Table 1) and body weight was measured weekly. Moreover, the implantation site was checked to confirm that the implant was still present and to check for any signs of inflammation or irritation. As some hens were observed to develop foot pad dermatitis (FPD) over the course of the experiment, special attention was paid to this disease and it was noted down weekly whether a hen was affected or not. Assessment of FPD was always performed by the same person who was blinded to the treatment. Affected hens were treated with antiseptics.

### Ultrasonography of Ovaries

**FIGURE 1:**
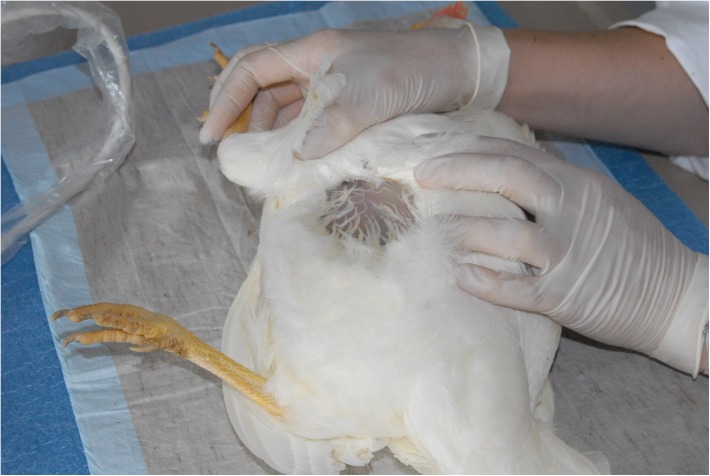
Area between the caudal rib and the dorsal column where the transducer was placed.

**FIGURE 2:**
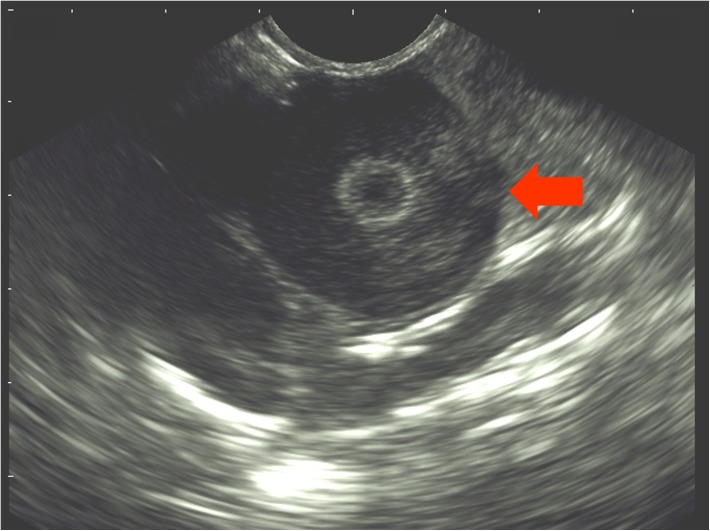
Sonogram of the ovary of a hen. The arrow marks an ovarian follicle.

Each hen was examined via ultrasonography weekly (Table 1) to check for ovarian follicles. The examination was conducted with the ultrasound system DUS 60 vet and the microconvex transducer C611-2 (both Edan Instruments GmbH, Shenzhen, China). The transducer was placed on the area between the vertebral column and the caudal rib (Figure 1). A frequency of 9.4 MHz was used and penetration depth varied between 39 and 58 mm. If present, follicles were visible as a round, anechoic zone with a smaller, round, hyperechoic zone in the middle (Figure 2). For each hen, the duration of effectiveness of the implant was assessed based on the number of weeks in which no follicles were detected by ultrasonography.

### Egg Yolk Staining and Egg Collection

As an additional control of egg production, egg yolks of treated and of control hens were stained in two different colors, based on a method described by Appleby and McRae (1983). Two different liposoluble dyes were used. Hens treated with deslorelin acetate were given Sudan Black B; control hens were given Oil Red O (both Sigma-Aldrich, St. Louis, United States). The dyes were administered weekly in gelatin capsules (Capsler, Stuhr, Germany) which contained 30 mg sugar and 30 mg dye. The liposoluble dye accumulates in the outer layer of all oocytes and can be seen in the egg yolk from the 2nd to the 10th day after administration.

Eggs were collected daily at 10 a.m. and cracked. The color of each egg yolk was noted and each egg could be related to control (red egg yolk) or treated hens (black egg yolk).

### Measurement of Estradiol-17ß Concentration in Plasma

Blood samples were taken weekly (Table 1) with twenty animals (five treated and five control hens of both “Adult” and “Juvenile”) being sampled each Tuesday, the remaining twenty animals each Wednesday. All blood samples were taken between 8 and 11 a.m. A maximum of 2 ml blood was taken from the ulnar vein. Immediately after sampling, blood samples were centrifuged at 3500 rpm at 4°C for 10 min. Plasma was stored at -20°C until further analysis.

Estradiol-17ß was measured in pg/ml using a commercial enzyme-linked immunosorbent assay (ELISA) kit (IBL International GmbH, Hamburg, Germany). A pool plasma sample was included on each kit together with the individual samples to calculate the inter-assay coefficient of variation which was 0.18. Each blood sample was measured in duplicate to calculate the intra-assay coefficient of variation. If the intra-assay coefficient was higher than ten, the measurement was repeated.

### Measurement of the Comb Size

From experimental week 13 on, the comb of each hen was photographed weekly with a digital reflex camera (Table 1). The hen was gently laid down on one side and the comb was placed on a small box (13.5 cm × 13.5 cm × 3.5 cm) to ensure it was plane. A ruler was placed beside the comb for scaling purposes.

The same person blindly evaluated all comb photos, using the image processing system AxioVision 4.8 (Carl Zeiss Microscopy GmbH, Jena, Germany). For each photo, a scale was generated using the ruler. Afterward, the outline of the comb was circumscribed and the size of its surface area was calculated by Axio Vision.

### Radiographic Examination of the Keel Bone

In order to detect any differences in keel bone damage between the treatments, all hens were radiographed in experimental weeks 7 and 15 (Table 1).

Digital, lateral radiographs were taken and evaluated as described previously (Eusemann et al., 2018) with 50.0 kV and at 2 mAs. The evaluation of all images was performed blindly by the same person and included the presence or absence of fractures and the measurement of deviations. The latter was used to calculate the proportion of deviated keel bone area (POD):

POD (%)=deviated area/keel bone surface area*100.

### Dissection

At the end of the experiment (experimental week 20), all hens were euthanized. Hens were stunned electronically and death was provoked by severing the jugular veins and carotid arteries. The hens of group “Adult” were 40 weeks old and the hens of group “Juvenile” were 33 weeks old at the time point of euthanasia (Table 1). The ovary, oviduct, heart, liver, spleen, intestine, gizzard, proventriculus, thyroid glands, lungs, kidneys, and brain were weighed and the relative organ weights were calculated by dividing organ weight by body weight. The length of the oviduct was measured. Before weighing the ovary, all large follicles were removed. Moreover, the implant was removed and the implantation site was checked once again for any signs of inflammation or irritation.

### Statistical Analysis

Differences between treated and control hens were analyzed separately for group “Adult” and group “Juvenile” due to different light programs and different experimental time periods between the groups. Data were analyzed using commercially available software (SAS 9.3, SAS Institute Inc., 2011).

For statistical analysis of the binary variables “Presence of follicles” and “Presence of FPD,” a linear logistic mixed model for repeated measurements (Littel et al., 2006) was applied. An analysis of covariance was performed for predicting the effect of age on “Presence of follicles” or “Presence of FPD.” Regression curves were fitted by considering age as a covariate term up to degree 4 of polynomials and the fixed effect of treatment (“treated” and “control”) as well as significant interactions between the main factor treatment and the covariate (age) up to degree 4 of polynomials. Least squares means were estimated on the logit scale to fulfill model assumptions and then back-transformed using the inverse link function to the original scale (probability to have follicles or FPD, respectively). *Post hoc* multiple comparisons of least squares means were performed using Tukey’s test.

Data of the numerical variables “Estradiol-17ß concentration,” “Body weight,” and “Comb size” were analyzed with a linear mixed model using the same factors as above but with underlying normal distribution. For analysis of estradiol-17ß concentrations, the fixed effects of treatment and age up to degree 4 of polynomials, their interaction up to degree 4 of polynomials, and the pre-treatment estradiol-17ß plasma concentrations were considered. For body weight development of the hens of group “Juvenile”, the interaction between treatment and age was only considered up to degree 2 of polynomials and for body weight development of group “Adult”, a second-degree polynomial was selected for the fixed regression term and the interaction as only these effects were shown to be significant. *Post hoc* multiple comparisons of least squares means were performed using Tukey’s test.

For analysis of POD in both groups and at both time points of data recording (experimental weeks 7 and 15), the fixed effect of treatment, the time point and its interaction were considered in the general linear model.

For statistical analysis of keel bone fractures, birds with one or multiple fractures were scored as 1 and those without any fracture as 0 at both time points. The effect of treatment on the binary outcome variable “fracture” was analyzed by means of one Chi-square test for each time point and group.

The relative weight to the body weight of the various organs at the end of the experiment was analyzed using a general linear model, considering only the fixed effect of the treatment in the statistical model as described above.

## Results

### General Health and Body Weight

No detectable adverse effects were found in any of the hens treated with a deslorelin acetate implant. Neither were there any signs of inflammation or irritation at the implantation site nor did treated hens show any negative alterations in health or behavior compared to control hens.

#### Group “Adult”

**FIGURE 3:**
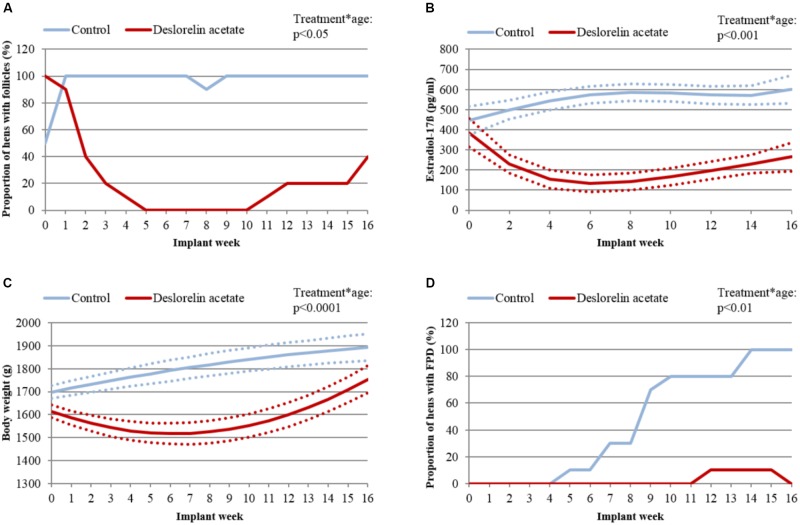
Hens with follicles, estradiol-17ß concentrations, body weight and hens with foot pad dermatitis (FPD) of group “Adult”. **(A)** Proportion of control (blue line) and treated hens (red line) with follicles on the ovary. **(B)** Least squares means (LSM; continuous lines) and upper and lower bounds of the 95% confidence interval (dotted lines) of estradiol-17ß in plasma in control (blue) and treated hens (red). Missing overlap of lines indicates a significant difference (*p* < 0.05) between groups. **(C)** LSM (continuous lines) and upper and lower bounds of the 95% confidence interval (dotted lines) of body weight of control (blue) and treated hens (red). Missing overlap of lines indicates a significant difference (*p* < 0.01) between groups. **(D)** Proportion of hens with FPD of control (blue) and treated hens (red).

Control hens of group “Adult” were heavier compared to treated hens throughout the experiment (treatment^∗^age: *p* < 0.0001; Figure 3C).

#### Group “Juvenile”

**FIGURE 4:**
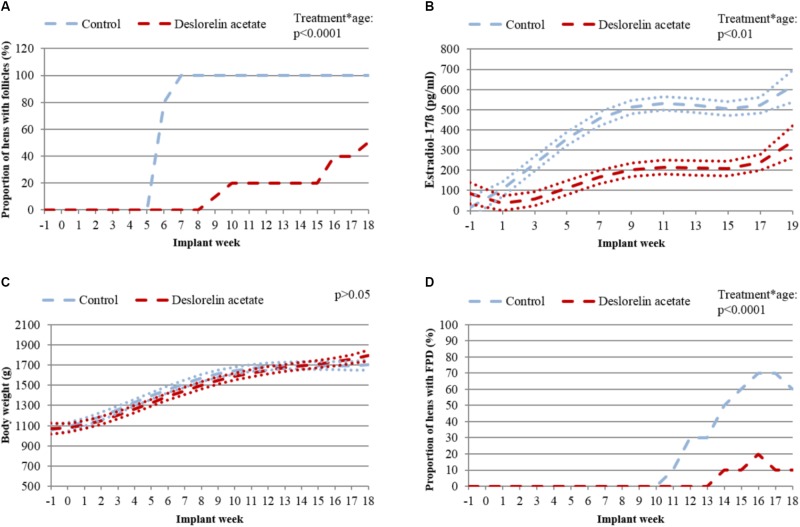
Hens with follicles, estradiol-17ß concentrations, body weight and hens with FPD of group “Juvenile”. **(A)** Proportion of control (blue dashed line) and treated hens (red dashed line) with follicles on the ovary. **(B)** LSM (dashed lines) and upper and lower bounds of the 95% confidence interval (dotted lines) of estradiol-17ß in plasma in control (blue) and treated hens (red). Missing overlap of lines indicates a significant difference (*p* < 0.05) between groups. **(C)** LSM (dashed lines) and upper and lower bounds of the 95 % confidence interval (dotted lines) of body weight of control (blue) and treated hens (red). Overlap of lines indicates no significant difference (*p* > 0.05) between groups. **(D)** Proportion of hens with FPD of control (blue dashed line) and treated hens (red dashed line).

In group “Juvenile”, no significant difference was found between body weight of control and treated hens (*p* = 0.22; Figure 4C).

### Follicles, Duration of Effectiveness, and Egg Production

#### Group “Adult”

In group “Adult”, proportion of hens with follicles was significantly higher in control compared to treated hens from implant week 2 to implant week 13 (treatment^∗^age: *p* < 0.05; Figure 3A). Follicles were detectable in all control hens from implant week 1 onwards. Only in implant week 8, one control hen did not show any detectable follicles. In all treated hens of group “Adult”, follicles were detectable prior to implantation. After implantation, the percentage of treated hens with follicles decreased continuously and between implant week 5 and implant week 10, none of the treated hens had follicles. From implant week 11 onwards, 4/10 treated hens developed follicles again. From implant week 14 onwards, treated and control hens of group “Adult” did not differ significantly in proportion of hens with follicles anymore (*p* > 0.05). The duration of effectiveness of the deslorelin implant showed interindividual differences. The shortest duration of effectiveness was 8 weeks in two treated hens while it was at least 16 weeks in one of the hens remaining without detectable follicles until the end of the experiment.

#### Group “Juvenile”

In none of the hens of group “Juvenile” follicles were detectable at the beginning of the study (Figure 4A). In control hens, ovarian follicles were first detected at implant week 6. Between implant week 7 and the end of the experiment (implant week 18), ovarian follicles were detectable in all control hens. 5/10 treated hens developed follicles between implant week 9 and the end of the study. The remaining five treated hens did not show follicles throughout the study. In group “Juvenile”, treated and control hens differed significantly in proportion of hens with follicles until the end of the study (treatment^∗^age: *p* < 0.0001).

#### Egg Yolk Staining and Egg Collection

The results of egg yolk staining and egg collection confirmed the findings of the ultrasonography in both groups (“Adult” and “Juvenile”): The number of eggs laid by control or treated hens corresponded to the number of hens in which follicles were detected via ultrasonography.

### Estradiol-17ß Concentration in Plasma

#### Group “Adult”

Estradiol-17ß plasma concentration was significantly higher in control compared to treated hens of group “Adult” from implant week 2 until the end of the experiment (treatment^∗^age: *p* < 0.001; Figure 3B). Both treated and control hens started with a high estradiol-17ß plasma concentration. While it remained at a high level, reaching more than 500 pg/ml estradiol-17ß in control hens, it decreased below 200 pg/ml in implant week 3 in treated hens. By implant week 6, estradiol-17ß concentration started to increase in treated hens but did not reach more than 300 pg/ml until the end of the experiment (implant week 16). Pre-treatment estradiol-17ß plasma concentrations did not affect estradiol-17ß concentrations after implantation (*p* = 0.71).

#### Group “Juvenile”

Both treated and control hens of group “Juvenile” started with a low concentration of estradiol-17ß (<100 pg/ml; Figure 4B). The concentration increased throughout the experiment in all hens. This increase in concentration was more pronounced and started earlier in control compared to treated hens. Consequently, higher concentrations of estradiol-17ß were found in control hens compared to treated hens from implant week 1 until the end of the experiment (treatment^∗^age: *p* < 0.01). Pre-treatment estradiol-17ß plasma concentrations did not affect estradiol-17ß concentrations after implantation (*p* = 0.75).

### Comb Size

The comb was significantly larger in control compared to treated hens in group “Adult” (LSM ± SE: 26.7 ± 1.52 cm^2^ vs. 6.66 ± 1.52 cm^2^; *p* < 0.0001) as well as in group “Juvenile” (27.05 ± 1.52 cm^2^ vs. 7.23 ± 1.52 cm^2^; *p* < 0.0001).

### Foot Pad Dermatitis

#### Group “Adult”

In group “Adult”, prevalence of FPD was significantly higher in control compared to treated hens from implant week 6 until the end of the experiment (treatment^∗^age: *p* < 0.01; Figure 3D). In implant week 5, 1/10 control hens was affected by FPD and, at the end of the experiment, all ten control hens were affected. In contrast, only 2/10 treated hens were affected by FPD throughout the entire experimental period. One of these two treated hens started laying eggs again 3 weeks prior to being affected by FPD.

#### Group “Juvenile”

In group “Juvenile”, prevalence of FPD was significantly higher in control compared to treated hens from implant week 11 until the end of the experiment (treatment^∗^age: *p* < 0.0001; Figure 4D). In implant week 11, 1/10 control hens was affected by FPD and the prevalence increased up to 7/10 control hens in implant weeks 16 and 17 before decreasing to 6/10 control hens in implant week 18. In contrast, only 2/10 treated hens were affected by FPD throughout the entire experimental period. Both of these two treated hens started laying eggs 5 weeks prior to being affected by FPD.

### Keel Bone Health

#### Group “Adult”

In group “Adult”, POD significantly increased from experimental week 7 (mean and standard error: 3.49 ± 0.5%) to experimental week 15 (4.44 ± 0.4%; *p* < 0.01). Moreover, deviated keel bone area was significantly larger in control (5.57 ± 0.6%) compared to treated hens (2.36 ± 0.6%; *p* < 0.01). None of the hens had a keel bone fracture in experimental week 7. In experimental week 15, the prevalence of keel bone fractures was significantly higher in control (4/10 hens) compared to treated hens (0/10 hens; *p* < 0.05).

#### Group “Juvenile”

In group “Juvenile”, no significant influence on POD was found for age (experimental week 7: 3.13 ± 0.53%, experimental week 15: 3.28 ± 0.4%; *p* = 0.76), treatment (control: 3.35 ± 0.57%, treated: 3.06 ± 0.57%; *p* = 0.72) or their interaction (*p* = 0.12). Similarly, no significant influence on prevalence of keel bone fractures was found for treatment (experimental week 7: *p* = 1; experimental week 15: *p* = 0.3). In experimental week 7, none of the hens had a keel bone fracture and in experimental week 15, one of the control hens but none of the treated hens had a keel bone fracture.

### Dissection

#### Group “Adult”

In group “Adult”, the relative weight of the following organs was significantly higher in control compared to treated hens (*p* < 0.01; Table 2): ovary, oviduct, liver, intestine, and kidneys. The relative weight of the spleen, in contrast, was significantly higher in treated compared to control hens (*p* < 0.05). No significant difference (*p* > 0.05) was found in the relative weight of the heart, gizzard, proventriculus, thyroid glands, lungs, and brain. The oviduct was significantly longer in control compared to treated hens (LSM ± SE: 61.9 ± 3.7 cm vs. 37.9 ± 3.7 cm; *p* < 0.001).

**Table 2:** Relative weight (% of body weight) of the organs of group “Adult” (left part) and group “Juvenile” (right part).

	Group “Adult”: Relative organ weight (% of body weight) (Least squares means ± Standard Error)		*P*-value	Group “Juvenile”: Relative organ weight (% of body weight) (Least squares means ± Standard Error)		*P*-value
	Control hens	Treated hens		Control hens	Treated hens	
Ovary	0.66 ± 0.05	0.26 ± 0.05	<0.0001	0.60 ± 0.07	0.38 ± 0.07	<0.05
Oviduct	3.55 ± 0.34	1.18 ± 0.32	<0.0001	3.43 ± 0.36	1.96 ± 0.38	<0.05
Liver	2.42 ± 0.07	1.80 ± 0.08	<0.0001	2.22 ± 0.12	1.76 ± 0.11	<0.05
Spleen	0.10 ± 0.01	0.14 ± 0.01	<0.05	0.11 ± 0.01	0.12 ± 0.01	0.26
Intestine	5.30 ± 0.14	4.73 ± 0.14	<0.01	5.30 ± 0.23	4.67 ± 0.23	0.06
Gizzard	1.77 ± 0.10	1.59 ± 0.10	0.21	1.85 ± 0.09	1.51 ± 0.09	<0.05
Proventriculus	0.36 ± 0.01	0.37 ± 0.01	0.6	0.37 ± 0.02	0.31 ± 0.02	<0.01
Thyroid glands	0.07 ± 0.03	0.01 ± 0.03	0.17	0.01 ± 0.001	0.01 ± 0.001	0.61
Lungs	0.40 ± 0.02	0.40 ± 0.02	0.77	0.38 ± 0.01	0.35 ± 0.01	0.12
Kidneys	0.72 ± 0.02	0.59 ± 0.02	<0.001	0.73 ± 0.03	0.54 ± 0.03	<0.001
Brain	0.18 ± 0.01	0.19 ± 0.01	0.38	0.18 ± 0.01	0.18 ± 0.01	0.57
Heart	0.37 ± 0.02	0.33 ± 0.02	0.15	0.37 ± 0.02	0.28 ± 0.02	<0.01

*P < 0.05 represent a significant difference between the least squares means of treated and control hens within one group (“Adult” or “Juvenile”, respectively).*

#### Group “Juvenile”

In group “Juvenile”, the relative weight of the following organs was significantly higher in control compared to treated hens (*p* < 0.05; Table 2): ovary, oviduct, liver, gizzard, proventriculus, kidneys, and heart. No significant difference (*p* > 0.05) was found in the relative weight of the spleen, intestine (*p* < 0.1), thyroid glands, lungs, and brain. The oviduct was significantly longer in control compared to treated hens (LSM ± SE: 62.4 ± 5.1 cm vs. 39.4 ± 5.1 cm; *p* < 0.01).

## Discussion

With the current study we have successfully established an animal model with non-laying and laying control hens which can be used in further studies to investigate the relationship between egg production and common diseases in laying hens as well as the underlying mechanisms.

One deslorelin acetate 4.7 mg implant has been shown to inhibit egg production both if implanted before and shortly after the onset of lay. Based on the aim of the study in which this animal model is intended to be used, researchers can decide whether to apply the implant before or after the onset of lay. In case of implantation after the onset of lay, laying hens have already developed all reproductive functions and thus have the same conditions as control hens. Implantation before the onset of lay might ensure that treated hens never lay any eggs throughout their lives. Further, this method can also be used to protract the onset of lay to investigate the relationship between the early onset of lay and different traits or diseases in laying hens. This could especially be interesting for studies concerning keel bone damage. Gebhardt-Henrich and Fröhlich (2015) found a negative correlation between the age of hens when laying their first egg and the probability of keel bone fracture presence at depopulation. The keel bone ossifies at about 35 weeks of age (FAWC, 2010) when laying hens have already been laying eggs for several weeks. Therefore, ossification might be disturbed by the competing demand for calcium to produce the egg shell, leading to a weak keel bone.

A very important finding of the current study is the relatively short duration of effectiveness of deslorelin acetate in laying hens. In dogs, the species the implant has been developed for, it is declared that Suprelorin^®^ 4.7 mg suppresses reproduction for 6 months, i.e., 24 weeks. However, in group “Adult” of the current study, the shortest duration of effectiveness was only 8 weeks in two treated hens and at only 14 weeks after implantation, treated and control hens did not differ significantly anymore in proportion of hens with follicles. These findings imply that when comparing non-laying and laying hens, implant application would have to be repeated the latest 14 weeks after initial implantation. However, these findings are not consistent with the study by Noonan et al. (2012) in which the same implant inhibited egg production for almost 26 weeks in laying hens. However, hens in the study by Noonan et al. were older than the hens in the present study which may explain this discrepancy. Moreover, the use of different layer lines may also result in different findings. However, we have no information which line Noonan et al. used in their study since this study was not published in a peer-reviewed journal, but as a scientific abstract (Noonan et al., 2012). Duration of effectiveness of deslorelin acetate, when implanted before the onset of lay, seemed to be increased since a statistically significant difference in proportion of hens with follicles between treated and control hens was still present at the end of the experiment, i.e., 18 weeks after implantation in group “Juvenile”. Due to different results between groups and studies and due to large interindividual differences in duration of effectiveness, it seems to be impossible to give a general recommendation concerning the interval after which a new deslorelin acetate implant should be administered in laying hens. In order to do so, long-term studies with hens of different layer lines that are treated at different ages are required.

In accordance to findings by Noonan et al. (2012), the deslorelin acetate implant was effective in all treated hens. In Japanese quail, however, deslorelin acetate did not inhibit egg production in some individuals (Petritz et al., 2013; Schmidt et al., 2013). Even after administration of two 4.7 mg implants or one 9.4 mg implant, some of the treated quail continued to lay eggs (Petritz et al., 2015). This indicates that the effect of deslorelin acetate largely differs between species and thus, results from one bird species cannot be readily transferred to another species.

In the current study, no placebo implant was given to control hens. This is in contrast to other studies which investigated the effect of sustained release deslorelin acetate implants on reproduction in different species (Trigg et al., 2001; Petritz et al., 2013, 2015; Schmidt et al., 2013; Summa et al., 2017). It is therefore possible that different findings in treated compared to control hens may be additionally due to the treatment procedure itself and not due to deslorelin acetate alone. However, as our results are very similar to results of studies with Japanese quail in which placebo implants were used (Petritz et al., 2013, 2015; Schmidt et al., 2013), it is more probable that differences were caused by deslorelin acetate. Nevertheless, a study investigating the effect of deslorelin acetate implants on egg production in laying hens including control hens receiving a placebo implant would help to strengthen our conclusions.

In both groups, “Adult” and “Juvenile”, no observable adverse effects of the implant were found. The injection site was devoid of signs of irritation or inflammation which indicates that the use of the implant is safe in laying hens. This is consistent with findings of other studies where deslorelin acetate implants were proven to be safe in different bird species (Cook and Riggs, 2007; Petritz et al., 2013). The only finding of the current study which may point toward an adverse effect was the lower body weight in treated compared to control hens of group “Adult”. However, this may also be explained by the weight of an inactive oviduct and ovary being much lower than the weight of an active oviduct and ovary. The difference in weight of these two organs alone is sufficient to explain the different body weight. Moreover, the relative weight of the digestive tract, the liver, and the kidneys was also higher in control compared to treated hens which may be explained by an increased activity of these organs in control hens, leading to the difference in body weight.

Some of our results on differences between treated and control hens in other traits may facilitate the time point determination of the implant’s effectiveness wearing off and its need to be replaced. Control hens displayed larger combs compared to treated hens. Thus, this characteristic may facilitate a quick estimation whether a hen lays eggs or not. Furthermore, estradiol-17ß plasma concentrations were lower in treated compared to control hens. Hence, repeated hormone concentration measurements may also serve as an indicator of implant effectiveness. However, in order to ensure if a hen is laying or not, ultrasonography of the ovary as well as egg yolk staining can be applied.

The findings on lower estradiol-17ß plasma concentrations in treated hens, which are in accordance with findings in other species (ferrets: Wagner et al., 2005; Japanese quail: Petritz et al., 2013), also have implications on further studies aiming at comparing laying with non-laying hens. As estradiol-17ß has an influence on several organs and mechanisms, it may be necessary to substitute this hormone in an animal model with deslorelin acetate depending on the hypothesis. For example, estradiol-17ß plays an important role in bone metabolism and bone diseases in laying hens as has been reviewed by Beck and Hansen (2004). In order to investigate the influence of egg production on bone health, it is therefore recommendable to substitute estradiol-17ß at least in a subgroup of the treated hens in order to compare laying and non-laying hens which have a similar estradiol-17ß plasma concentration.

The decreased keel bone deviations and fractures in treated compared to control hens of group “Adult” as well as the incidental finding of decreased FPD prevalence in treated compared to control hens of both groups indicate that egg production may be related to these diseases. Concerning keel bone damage, our findings in group “Adult” support the hypothesis that the high demand for calcium to produce the egg shell leads to weaker bones. In contrast, we did not find any differences between treated and control hens in group “Juvenile”. This may be explained by the relatively young age in which these hens were radiographed (20th and 28th week of age). Keel bone damage has been shown to increase with age (Käppeli et al., 2011a; Heerkens et al., 2016; Eusemann et al., 2018). Consequently, we may have detected differences between control ( = laying) and treated ( = non-laying) juvenile hens later in life, i.e., if we had kept and radiographed the hens at later ages. In order to gain more insight into the etiology of keel bone fractures and deviations and the role of egg production, a study comparing keel bones of laying and non-laying hens throughout the entire laying period would be required. Concerning FPD, almost all affected hens were control hens or treated hens which had started laying eggs again. This finding suggests that non-laying hens may display a more active immune system which is in accordance with the higher relative weight of the spleen, an important organ of the immune system, in treated compared to control hens. Differences in the immune system between laying and non-laying hens could be explained by different plasma concentrations of estradiol-17ß and possibly other gonadal steroids as these have been shown to regulate the immune system (reviewed by Grossman, 1985). However, to fully understand this incidental finding, the relationship between egg production, estradiol-17ß plasma concentrations, FPD, and the immune system needs to be investigated in more detail.

## Conclusion

We present a valid animal model with non-laying and laying control hens which can be used to investigate the relationship between egg production and different diseases in laying hens. This model has been achieved by administration of one 4.7 mg deslorelin acetate implant per hen. However, based on our results, duration of effectiveness in laying hens seems to be much shorter than previously reported.

Furthermore, we have shown differences between treated and control hens in other traits such as comb size and estradiol-17ß concentrations. Lastly, our findings indicate that keel bone fractures and deviations as well as FPD may be related to egg production in laying hens.

## Data Availability Statement

The raw data supporting the conclusions of this manuscript will be made available by the authors, without undue reservation, to any qualified researcher.

## Author Contributions

BE, LS, and SP conceived and designed the experiments. BE and A-KR performed the experiments. AS, BE, and AP analyzed the data. BE wrote the original draft of the manuscript. AS wrote the section on statistical methods. AP, SP, LS, CT-R, and AS reviewed and edited the original draft of the manuscript. BE visualized the data. All authors read and approved of the final manuscript.

## Conflict of Interest Statement

The authors declare that the research was conducted in the absence of any commercial or financial relationships that could be construed as a potential conflict of interest.
